# Supplementary material for: Quantification of Letrozole, Palbociclib, Ribociclib, Abemaciclib, and Metabolites in Volumetric Dried Blood Spots: Development and Validation of an LC-MS/MS Method for Therapeutic Drug Monitoring
Source: Int J Mol Sci. 2024 Sep 27;25(19):10453. doi: 10.3390/ijms251910453 (PMC11476960; doi:10.3390/ijms251910453)
Supplement: Supplementary file 1 [file ijms-25-10453-s001.zip › Supporting Information revised.pdf]

Table S1. Within-run precision and accuracy data for letrozole, abemaciclib, M2, M20, palbociclib, and ribociclib.

Analyte	Nom conc. (ng/mL)	Run 1 Mean ±SD (ng/mL)	Run 1 CV%	Run 1 Acc%	Run 2 Mean ±SD (ng/mL)	Run 2 CV%	Run 2 Acc%	Run 3 Mean ±SD (ng/mL)	Run 3 CV%	Run 3 Acc%
letrozole	6.0	6.0±0.3	5.4	100	6.5±0.2	3.5	108	5.8±0.2	3.5	97
	16.1	15.3±0.5	3.1	95	16.0±0.3	2.1	99	15.0±0.5	3.3	93
	92.0	90.9±2.1	2.4	99	90.9±2.1	2.3	99	89.0±1.5	1.7	97
	230.0	225.0±3.7	1.7	98	232.8±3.3	1.4	101	222.8±2.9	1.3	97
abemaciclib	40.0	37.8±1.8	4.7	95	43.4±2.0	4.6	108	39.0±1.2	3.1	98
	93.0	91.8±6.6	7.2	99	98.8±2.5	2.5	106	90.3±3.9	4.3	97
	248.0	236.6±4.7	2.0	95	244.0±7.7	3.2	98	241.8±4.7	1.9	98
	620.0	625.2±20.2	3.2	101	639.0±17.8	2.8	103	609.6±26.6	4.4	98
M2	20.0	18.5±1.5	8.1	93	22.4±1.5	6.5	112	19.8±1.4	7.2	99
	46.5	42.3±0.9	2.2	91	47.3±1.3	2.7	102	44.0±1.1	2.6	95
	124.0	112.6±4.8	4.3	91	121.8±0.8	0.7	98	118.4±5.9	4.9	95
	310.0	295.4±20.6	7.0	95	314.8±9.0	2.9	102	303.2±10.6	3.5	98
M20	20.0	21.5±3.2	14.9	107	22.0±1.1	4.9	110	29.8±1.2	6.0	99
	46.5	45.2±2.6	5.8	97	48.8±2.3	4.8	105	43.7±3.5	8.0	94
	124.0	117.8±3.1	2.6	95	120.8±5.1	4.2	97	118.6±6.2	5.2	96
	310.0	319.6±9.3	2.9	103	312.6±6.8	2.2	101	285.8±13.3	4.7	92
palbociclib	6.0	5.4±0.4	7.7	90	6.6±0.4	5.6	109	5.7±0.3	5.6	94
	16.1	16.1±0.7	4.2	100	15.7±0.7	4.6	98	14.8±0.3	2.3	92
	92.0	93.7±1.1	1.2	102	91.2±2.6	2.8	99	87.8±1.3	1.5	95
	230.0	237.2±4.4	1.8	103	227.6±3.8	1.7	99	210.8±6.3	3.0	92
ribociclib	120.0	123.0±4.3	3.5	103	127.2±3.6	2.9	106	103.8±4.0	3.9	87
	315.0	299.4±11.8	4.0	95	308.8±9.9	3.2	98	296.4±3.9	1.3	94
	1800.0	1788.0±39.0	2.2	99	1774.0±43.4	2.4	99	1786.0±62.3	3.5	99
	4500.0	4502.0±150.4	3.3	100	4408.0±154.5	3.5	98	4256.0±109.2	2.6	95

Table S2. Precision and accuracy of the calibration curves.

Analyte	Nominal conc. (ng/mL)	Mean ± SD	CV%	Acc%
abemaciclib	40.0	49.7±1.6	4.0	99
	80.0	81.1±1.9	2.4	101
	160.0	161.2±7.8	4.8	101
	240.0	239.0±12.6	5.3	100
	320.0	323.0±11.7	3.6	101
	480.0	472.0±8.6	1.8	98
	640.0	635.4±17.9	2.8	99
	800.0	804.2±23.1	2.9	101
M2	20.0	19.9±0.9	4.7	100
	40.0	40.5±2.3	5.6	101
	80.0	79.4±5.9	6.7	99
	120.0	119.1±5.9	5.0	99
	160.0	162.0±7.5	4.6	101
	240.0	235.8±8.4	3.6	98
	320.0	317.8±10.4	3.3	99
	400.0	407.3±14.4	3.5	102
M20	20.0	20.0±0.7	3.3	100
	40.0	39.9±2.9	7.3	100
	80.0	79.8±4.0	5.0	100
	120.0	118.0±10.8	9.1	98
	160.0	163.8±7.2	4.4	102
	240.0	233.9±5.8	2.5	97
	320.0	318.0±12.2	3.8	99
	400.0	404.1±16.1	4.0	101
letrozole	6.0	6.0±0.1	1.7	99
	12.0	12.2±0.4	3.6	101
	60.0	59.6±1.3	2.2	99
	120.0	120.6±2.5	2.1	101
	150.0	147.8±1.3	0.9	99
	180.0	178.2±2.5	1.4	99
	240.0	240.0±4.7	2.0	100

	300.0	305.2±5.7	1.9	102
palbociclib	6.0	5.9±0.3	5.3	99
	12.0	12.3±0.7	5.8	102
	60.0	59.8±2.1	3.5	100
	120.0	119.9±3.1	2.6	100
	150.0	147.5±3.8	2.6	98
	180.0	178.3±4.7	2.6	99
	240.0	241.0±6.5	2.7	100
	300.0	305.1±8.1	2.7	102
ribociclib	120.0	118.7±4.2	3.6	99
	240.0	244.5±12.5	5.1	102
	1200.0	1235.0±36.6	3.0	103
	2400.0	2420.0±80.3	3.3	101
	3000.0	2987.0±45.7	1.5	100
	3600.0	3546.0±86.2	2.4	99
	4800.0	4698.0±67.6	1.4	98
	6000.0	5961.0±224.5	3.8	99

Table S3. Recovery of letrozole, abemaciclib, M2, M20, palbociclib, and ribociclib in DBS samples.

Analyte	Nominal conc. (ng/mL)	Recovery (%) ± SD	Recovery CV(%)
letrozole	16.1	93.4±11.5	12.3
	230.0	91.4±4.8	5.2
abemaciclib	93.0	89.5±6.5	7.3
	620.0	87.6±4.0	4.0
M2	46.5	86.2±6.2	7.2
	310.0	84.9±3.5	4.1
M20	46.5	88.1±6.8	7.7
	310.0	88.0±5.4	6.2
palbociclib	16.1	81.1±6.2	7.6
	230.0	80.9±3.9	4.8
ribociclib	315.0	80.9±5.5	6.8
	4500.0	82.9±3.6	4.3

Table S4. Accuracy, precision and matrix effect (ME) of letrozole, abemaciclib, M2, M20, palbociclib, and ribociclib in DBS samples.

Analyte	Nominal conc. (ng/mL)	Mean ±SD (ng/mL)	Acc%	CV%	ME % ± SD	ME CV%
letrozole	16.1	15.4±0.4	96	2.9	0.93±0.08	8.4
	230.0	222.9±4.9	97	2.2	0.94±0.04	4.4
abemaciclib	93.0	89.6±4.1	96	4.5	0.96±0.08	8.5
	620.0	606.3±30.6	98	5.0	0.99±0.07	6.9
M2	46.5	44.3±2.1	95	4.7	1.00±0.07	6.8
	310.0	304.0±20.6	98	6.8	1.07±0.06	5.6
M20	46.5	44.9±3.0	96	6.7	0.96±0.08	8.9
	310.0	306.4±18.6	99	6.1	1.01±0.07	7.2
palbociclib	16.1	16.0±0.7	99	4.5	0.89±0.04	4.5
	230.0	226.0±6.9	98	3.1	0.88±0.02	2.0
ribociclib	315.0	307.0±10.3	97	3.4	0.88±0.04	5.0
	4500.0	4334.8±139.7	96	3.2	0.90±0.02	1.9

Table S5. Stability of letrozole, abemaciclib, M2, M20, palbociclib, and ribociclib in autosampler after extraction.

Analytes	Nominal conc. (ng/mL)	5 days in autosampler (5°C)			11 months in Sicco (20 °C, humidity < 35%)		
		Mean ± SD	Acc%	CV%	Mean ± SD	Acc%	CV%
letrozole	16.1	15.8±0.6	98	3.5	15.8±0.5	98	2.8
	230.0	231.3±0.6	101	0.2	224.3±1.5	98	0.7
abemaciclib	93.0	91.8±2.9	99	3.2	94.0±0.6	101	0.7
	620.0	639.3±11.7	103	1.8	615.0±10.6	99	1.7
M2	46.5	48.2±0.4	104	0.9	45.5±2.3	98	5.0
	310.0	327.3±14.5	106	4.4	297.0±5.3	96	1.8
M20	46.5	45.4±2.6	98	5.7	44.2±0.7	95	1.6
	310.0	316.3±14.3	102	4.5	287.7±16.2	93	5.6

palbociclib	16.1	15.6±1.6	97	10.2	14.4±0.3	89	1.8
	230.0	229.3±8.0	100	3.5	209.0±5.3	91	2.5
ribociclib	315.0	324.3±11.7	103	3.6	270.3±10.7	86	4.0
	4500.0	4490.0±62.4	100	1.4	4116.7±121.0	91	2.9

Table S6. Stability of letrozole, abemaciclib, M2, M20, palbociclib, and ribociclib for home sampling conditions.

Analytes	Nominal conc. (ng/mL)	2 weeks in plastic bags + silica gel (part I)			7 weeks in Sicco (20 °C, humidity < 35%) (part II)		
		Mean ± SD	Acc%	CV%	Mean ± SD	Acc%	CV%
letrozole	16.1	15.1±0.1	94	0.8	15.0±0.5	99	3.5
	230.0	222.3±1.5	97	0.7	214.7±3.8	97	1.8
abemaciclib	93.0	90.7±7.3	98	8.1	86.0±3.7	95	4.3
	620.0	581.7±28.7	94	4.9	591.0±13.1	102	2.2
M2	46.5	46.9±3.1	101	6.5	41.5±0.2	88	0.5
	310.0	296.3±13.6	96	4.6	294.0±6.1	99	2.1
M20	46.5	42.9±3.3	92	7.7	42.9±0.4	100	0.9
	310.0	283.0±13.7	91	4.9	299.7±12.7	106	4.2
palbociclib	16.1	14.6±0.5	91	3.5	14.4±0.5	98	3.3
	230.0	210.3±6.7	91	3.2	203.3±4.5	97	2.2
ribociclib	315.0	282.3±6.7	90	2.4	268.0±7.0	95	2.6
	4500.0	4233.3±47.3	94	1.1	3870.0±45.8	91	1.2

Table S7. Patient characteristics at recruitment.

Patient characteristics		number
Total patients		28
Gender. female		100%
Median age (range)		55 (36-81) years
Therapeutic scheme	abemaciclib (100 mg/die) + letrozole (2.5 mg/die)	1
	abemaciclib (150 mg/die) + letrozole (2.5 mg/die)	2
	abemaciclib (200 mg/die) + letrozole (2.5 mg/die)*	4
	abemaciclib (300 mg/die) + letrozole (2.5 mg/die)**	13
	palbociclib (100 mg/die) + letrozole (2.5 mg/die)	1
	palbociclib (125 mg/die) + letrozole (2.5 mg/die)	2
	ribociclib (400 mg/die) + letrozole (2.5 mg/die)	2
	ribociclib (600 mg/die) + letrozole (2.5 mg/die)	3

* exemestane (25 mg/die) in 1 patients; **fulvestrant (500 mg IM monthly) in 4 patients.

Table S8. Stock and working solution (SS, WS) concentrations of the calibrators (A-H) and QCs (H-L). WS were diluted in MeOH.

solution	Abemaciclib	M2	M20	Palbociclib	Ribociclib	Letrozole
SS (mg/mL)	0.5 (in DMSO)	0.5 (in HCl 0.01 M)	0.5 (in DMSO)	0.2 (in DMSO)	2 (in MeOH)	1 (in MeOH)
A	16000.0	8000.0	8000.0	6000.0	120000.0	6000.0
B	12800.0	6400.0	6400.0	4800.0	96000.0	4800.0
C	9600.0	4800.0	4800.0	3600.0	72000.0	3600.0
D	6400.0	3200.0	3200.0	3000.0	60000.0	3000.0
E	4800.0	2400.0	2400.0	2400.0	48000.0	2400.0
F	3200.0	1600.0	1600.0	1200.0	24000.0	1200.0
G	1600.0	800.0	800.0	240.0	4800.0	240.0
H	800.0	400.0	400.0	120.0	2400.0	120.0
QCL	1860.0	930.0	930.0	322.0	6300.0	322.0
QCM	4960.0	2480.0	2480.0	1840.0	36000.0	1840.0
QCH	12400.0	6200.0	6200.0	4600.0	90000.0	4600.0

Table S9. Concentrations of the calibrators and QCs DBS samples expressed in ng/mL.

	Abemaciclib	M2	M20	Palbociclib	Ribociclib	Letrozole
A	800.0	400.0	400.0	300.0	6000.0	300.0
B	640.0	320.0	320.0	240.0	4800.0	240.0
C	480.0	240.0	240.0	180.0	3600.0	180.0
D	320.0	160.0	160.0	150.0	3000.0	150.0
E	240.0	120.0	120.0	120.0	2400.0	120.0
F	160.0	80.0	80.0	60.0	1200.0	60.0
G	80.0	40.0	40.0	12.0	240.0	12.0
H	40.0	20.0	20.0	6.0	120.0	6.0
QCL	93.0	46.5	46.5	16.1	315.0	16.1
QCM	248.0	124.0	124.0	92.0	1800.0	92.0
QCH	620.0	310.0	310.0	230.0	4500.0	230.0

Table S10. Compound-dependent parameters and ion transitions of each analyte and IS used for mass spectrometry.

	Precursor Ion			Product Ion		
**Analyte**	**Q1 (amu)**	**DP (volts)**	**EP (volts)**	**Q3 (amu)**	**CE (volts)**	**CXP (volts)**
letrozole	284	-90	-10	242	-30	-10
				215	-45	-10
13C2,15N2-letrozole	288	-50	-10	246	-30	-10
Abemaciclib	507	140	13	393	33	20
				351	60	16
M20	523	140	12	409	30	22
				379	54	21
M2	479	120	7	393	30	22
				245	75	17
Palbociclib	448	130	10	380	40	10
				337	53	10
Ribociclib	435	110	10	322	63	10
				367	55	10
D8-abemaciclib	515	135	9	393	35	25
D8-palbociclib	456	130	10	388	40	10
D6-ribociclib	441	110	10	373	38	10

Table S11. Source-dependent parameters used for mass spectrometry. The source-dependent parameters were optimized based on the abemaciclib metabolites, M2 and M20, which had a lower signal than the other compounds.

Ion source	Turbo Spray
Polarity	Negative / positive
Curtain gas (CUR) (nitrogen)	30 psi
Collision gas (CAD)	medium intensity
Ion Spray voltage (IS)	-1500 V (letrozole and $^{13}C_2$,$^{15}N_2$-letrozole) / 5000 V
Temperature (TEM)	500 °C
Ion source gas 1 (GS1)- nebulizer gas pressure	30 psi
Ion source gas 2 (GS2)- heater gas pressure	60 psi
