# Supplementary figures and images for: Quantification of Letrozole, Palbociclib, Ribociclib, Abemaciclib, and Metabolites in Volumetric Dried Blood Spots: Development and Validation of an LC-MS/MS Method for Therapeutic Drug Monitoring
Source: Int J Mol Sci. 2024 Sep 27;25(19):10453. doi: 10.3390/ijms251910453 (PMC11476960; doi:10.3390/ijms251910453)

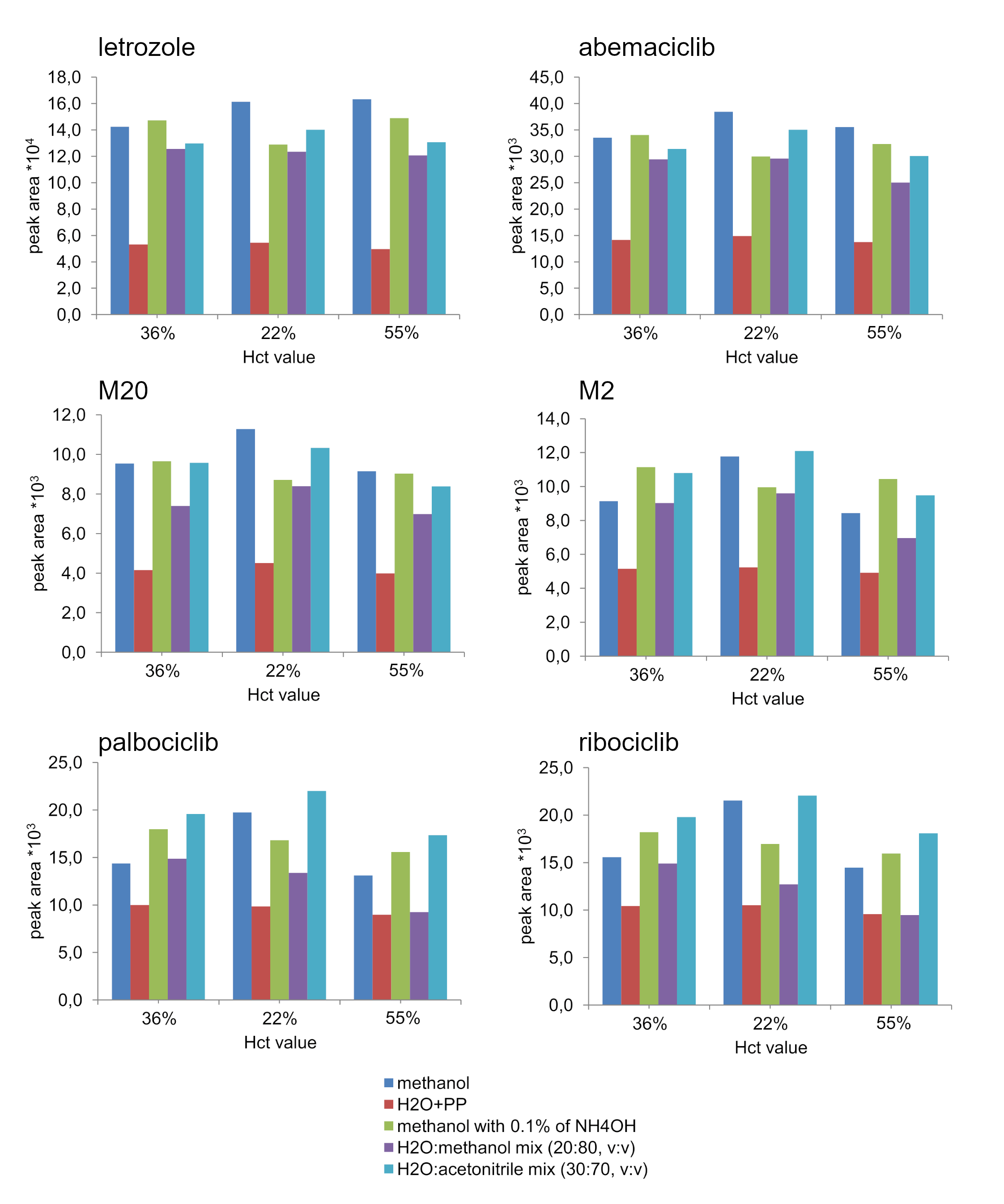

Supplement: Supplementary file 1 [file ijms-25-10453-s001.zip › Figure S1.tif]

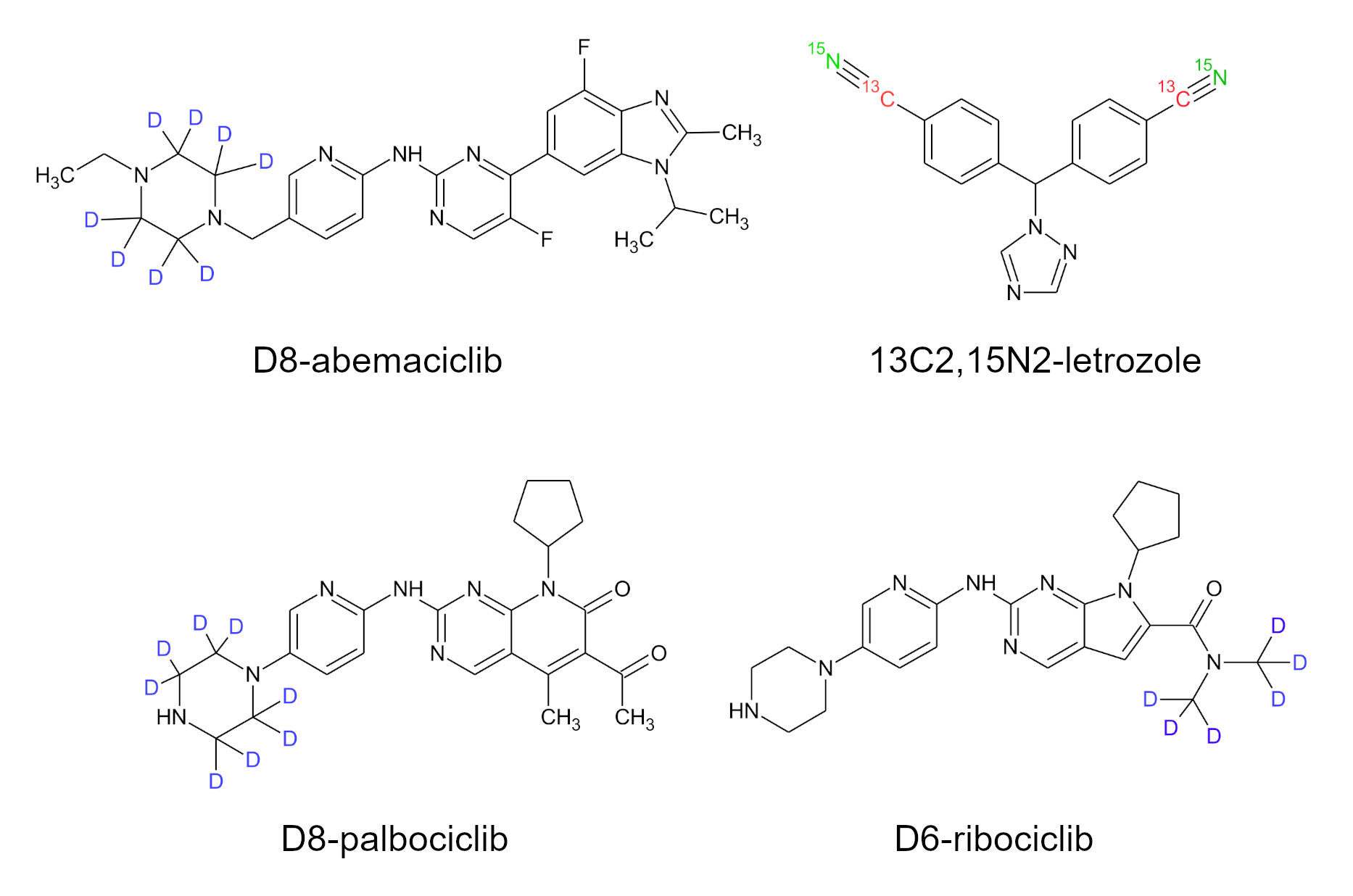

Supplement: Supplementary file 1 [file ijms-25-10453-s001.zip › Figure S2.tif]
